# Evidence based post-operative treatment of flexor tendons

**DOI:** 10.1186/1753-6561-9-S3-A109

**Published:** 2015-05-19

**Authors:** Sarah Ewald

**Affiliations:** 1 City Handtherapie, Zurich, 8006, Switzerland

## 

Flexor Tendon injuries require surgical repair and intensive therapy post-operatively for optimal outcomes. “The repair technique is important but it is the way the tendon is managed afterwards that determines the outcome” ([1], page 112). Multiple protocols exist for the post-operative rehabilitation of tendon injuries. Currently four main options for post-operative treatment are utilized: immobilization, early passive mobilization, early active-passive mobilization and earlyactive mobilization. Early protected movement has been shown to decrease adhesion formation and stimulate tendon healing [2]. It is well established that protected early movement of the tendon results in a better outcome than immobilization [3-5]. There is less risk of tendon rupture with early passive motion protocols, but an increased risk for decreased range of motion in the final outcome, in comparison to early active motion protocols [6].

“The goal of tendon repair and rehabilitation is to achieve a normally gliding and functioning tendon.”([1], p 115). To achieve this goal the surgeon, the therapist and the patient must work closely together. The surgeon must create a repair that is strong enough to withstand the rehabilitation phase. The therapist must have a thorough understanding of: tendon anatomy, the mechanical limitations of the repair, the healing process and the biomechanical forces at work during the healing process. Additionally the therapist must understand the tendon’s response to the chosen treatment and continually adjust the treatment accordingly. The patient too must participate in the treatment; this requires that the patient receives sufficient instruction that allows him or her to understand the injury and participate in the subsequent rehabilitation process.

In many clinics, flexor tendon rehabilitation is protocol driven; a standard post-operative protocol is implemented routinely for flexor tendon injury cases. This approach may work well when the patient meets the standard inclusion criteria. However when the patient does not meet the criteria for the prescribed protocol for post-operative treatment, treatment of acute flexor tendon injuries can be a challenge and if not adjusted for may result in less than optimal outcomes. “Functional outcomes do not depend on following a prescribed protocol, but on progressing each patient individually with the available evidence-based information and on observation of the individual’s healing process” [7]. There is ample evidence available to guide therapists and physicians in clinical decision making when patient characteristics do not fit the protocol. A review of evidence that supports and guides the therapist’s clinical decision making in rehabilitation of the flexor tendon will be presented. Options for clinical situations such as: delayed initiation of post-operative treatment, concomitant injuries and or conditions that require rest vs. movement, patients that cannot follow complex treatment programs and other clinical scenarios that require an adapted approach to obtain an optimal outcome, will be discussed.
